# Movement and conformity interact to establish local behavioural traditions in animal populations

**DOI:** 10.1371/journal.pcbi.1006647

**Published:** 2018-12-20

**Authors:** Marius Somveille, Josh A. Firth, Lucy M. Aplin, Damien R. Farine, Ben C. Sheldon, Robin N. Thompson

**Affiliations:** 1 Edward Grey Institute, Department of Zoology, University of Oxford, Oxford, United Kingdom; 2 Max Planck–Yale Center for Biodiversity Movement and Global Change, Department of Ecology and Evolutionary Biology, Yale University, New Haven, CT, United States of America; 3 Merton College, University of Oxford, Oxford, United Kingdom; 4 Cognitive and Cultural Ecology Research Group, Max Planck Institute for Ornithology, Radolfzell, Germany; 5 Department of Collective Behaviour, Max Planck Institute for Ornithology, Konstanz, Germany; 6 Chair of Biodiversity and Collective Behaviour, Department of Biology, University of Konstanz, Konstanz, Germany; 7 Department of Zoology, University of Oxford, Oxford, United Kingdom; 8 Mathematical Institute, University of Oxford, Radcliffe Observatory Quarter, Oxford, United Kingdom; 9 Christ Church, University of Oxford, St. Aldates, Oxford, United Kingdom; UNITED KINGDOM

## Abstract

The social transmission of information is critical to the emergence of animal culture. Two processes are predicted to play key roles in how socially-transmitted information spreads in animal populations: the movement of individuals across the landscape and conformist social learning. We develop a model that, for the first time, explicitly integrates these processes to investigate their impacts on the spread of behavioural preferences. Our results reveal a strong interplay between movement and conformity in determining whether locally-variable traditions establish across a landscape or whether a single preference dominates the whole population. The model is able to replicate a real-world cultural diffusion experiment in great tits *Parus major*, but also allows for a range of predictions for the emergence of animal culture under various initial conditions, habitat structure and strength of conformist bias to be made. Integrating social behaviour with ecological variation will be important for understanding the stability and diversity of culture in animals.

## Author summary

In many animal species, the social transmission of information is important and can lead to the emergence of behavioural traditions. However, how ecological and social processes together influence information transmission and its consequences for animal culture, particularly across space, remains largely unknown. We developed a spatially-explicit model examining the spread of behavioural preference through a population, which integrates two key processes: the movement of individuals in the landscape, and social learning with conformity (the disproportionate likelihood of adopting the behavioural trait that is most common locally). The model can replicate a real-world cultural diffusion experiment in great tits *Parus major*. It is also general, and allows us to make predictions about the emergence of animal cultures in a range of different ecological and social scenarios, including habitats with different states of fragmentation, species with varying movement patterns, and different strengths of conformity in the transmission of behaviour. Our results reveal a strong interplay between ecological and social processes (in this case, movement and conformity) for determining whether or not traditions establish within a population. If traditions do emerge, then these can either be local or global, depending on the relative strength of conformity compared to movement.

## Introduction

The social transmission of information plays a central role in the lives of many animal species [1–3]. Social learning via observation of, or interaction with, other individuals is an efficient mechanism for acquiring information about the environment, leading to adaptive adjustments of behavioural responses [4,5]. The transmission of information through social networks can lead to the emergence of regional variations in behaviour that are stable through time (called local traditions; [6–10]). Animal culture is defined as “group-typical behavioural patterns, shared by members of animal communities, that are to some degree reliant on socially learned and transmitted information” [8]. However, we still have little mechanistic understanding of the conditions under which local cultures can emerge. Understanding how ecological, cognitive and social processes determine the spread of information between individuals in wild populations is crucial if we want to discern the conditions under which information spreads and local traditions emerge.

A key ecological process that is likely to affect the spread of information is movement. First, movement of animals between discrete groups or sub-populations is expected to accelerate information spread across the whole population. Second, moving individuals can potentially import different behaviours into local groups or sub-population [11,12]. How individuals move in a landscape, itself likely to be influenced by a range of factors such as habitat configuration [13,14] and demography [15], is therefore likely to shape the dynamics of behaviours in natural populations.

One of the main socio-cognitive factors thought to affect the emergence of culture is conformity [16]. Conformist social learning is here defined as positive frequency-dependent copying, where individuals are disproportionately likely to adopt the most common behavioural trait [17]. Importantly, if individuals exhibit conformist learning, a single socially-learnt behavioural variant might become fixed in a group, remain stable over time and be resistant to invasion by alternative variants, thus leading to the establishment and persistence of group-specific traditions. Several studies support the hypothesis that conformity plays an important role in the establishment and stability of local traditions in various animal species [11,12,18,19]. However, we lack an understanding of when conformist learning is needed to generate established traditions. The interplay between social learning biases such as conformity, and the ecological factors that determine the context in which such learning takes place, could potentially produce varying outcomes, but has rarely been studied.

Here, we investigate how movement and conformity interact to shape the spread of socially-transmitted information and the establishment of local traditions in animal populations. We first develop a theoretical spatially-explicit model of the spread of behavioural preference in which a problem (representing a novel foraging resource) can be solved in one of two ways. A “behavioural preference” refers to the solution used by a given solver (i.e. an individual who knows how to solve the problem) at a given time. The population is assumed to be composed of several spatially distinct sub-populations, and each individual is either unable to solve the problem, or solves the problem with a preference for one of the two solutions. Individuals can learn the behaviour from each other, with a conformist bias guiding which of the two preferences they learn. Individuals can also move between sub-populations. We then use this model to investigate the conditions under which local cultural traditions emerge. We initially consider simple scenarios in which there are only one, two or three sub-populations. In addition, we test the model’s ability to replicate a real-world cultural diffusion experiment [11], in which alternative novel foraging techniques–consisting of opening a bi-directional door puzzle-box by sliding it either left or right to access food–were introduced in several wild sub-populations of great tits *Parus major*. The spread of these foraging behaviours in the population was monitored, revealing that the behaviour was socially transmitted with a significant conformist bias, and that local foraging traditions established within the population. As we show, our modelling approach can recreate the empirical results of the experiment in this wild population [11], and predict the conditions under which such local traditions are likely to establish and persist in the population.

## Results

Our model integrates the movement of individuals across the landscape and the social process of transmission of information between individuals, including a conformist bias. The spread of behavioural preferences for solutions to a problem (solutions s_1_ and s_2_) occurs in an environment with a metapopulation structure composed of connected sub-populations (see details in Materials and Methods). These sub-populations are composed of naïve individuals (i.e. individuals who do not know how to perform the behaviour) and knowledgeable individuals (i.e. individuals who have either learnt one of the solutions from other individuals, or who knew that behaviour from the start, the latter being called “innovators”). At the start of each numerical solution of the model (hereafter, “simulation”), innovators are introduced into the system (which otherwise only comprised of naïve individuals), with different behavioural preferences in each sub-population, and the spread of behavioural preferences is then simulated.

### Single population analysis

The dynamics of acquisition of behavioural preferences within a single mixed population greatly depends on the conformist learning function, represented by parameter *λ* (see Materials and Methods; S1 Fig and S2 Fig). When *λ* < 1, indicating preferential copying of the rarest behavioural variant relative to its prevalence, the system stabilises towards equal proportions of solvers using solutions s_1_ and s_2_ (S2 Fig). When *λ* = 1, indicating copying proportional to the prevalence of the behavioural variant, solvers using solutions s_1_ and s_2_ coexist in the population, but their respective proportions depend on the initial conditions (i.e. proportions of seeded individuals ‘trained’ to solve the problem using each solution at the start of the simulation; S2 Fig). When *λ* > 1, corresponding to a conformity bias in the learning process, unless the same number of individuals using solutions s_1_ and s_2_ are initially present in the population (in which case they both end up comprising 50% of the population at the end of the simulation), there are two stable attraction points: solution s_1_ takes over (i.e. 100% of the individuals in the population use it at the end of the simulation) if the initial population consists of more trained innovators using this solution, or solution s_2_ takes over if initially the population consists of more trained innovators using this solution (S2 Fig). These results indicate that *λ* ≥ 1 is necessary to have stable locally dominant behaviours when both behavioural preferences initially exist in the population. We therefore restricted further model exploration to *λ* ≥ 1.

### The baseline model

**Fig 1:**
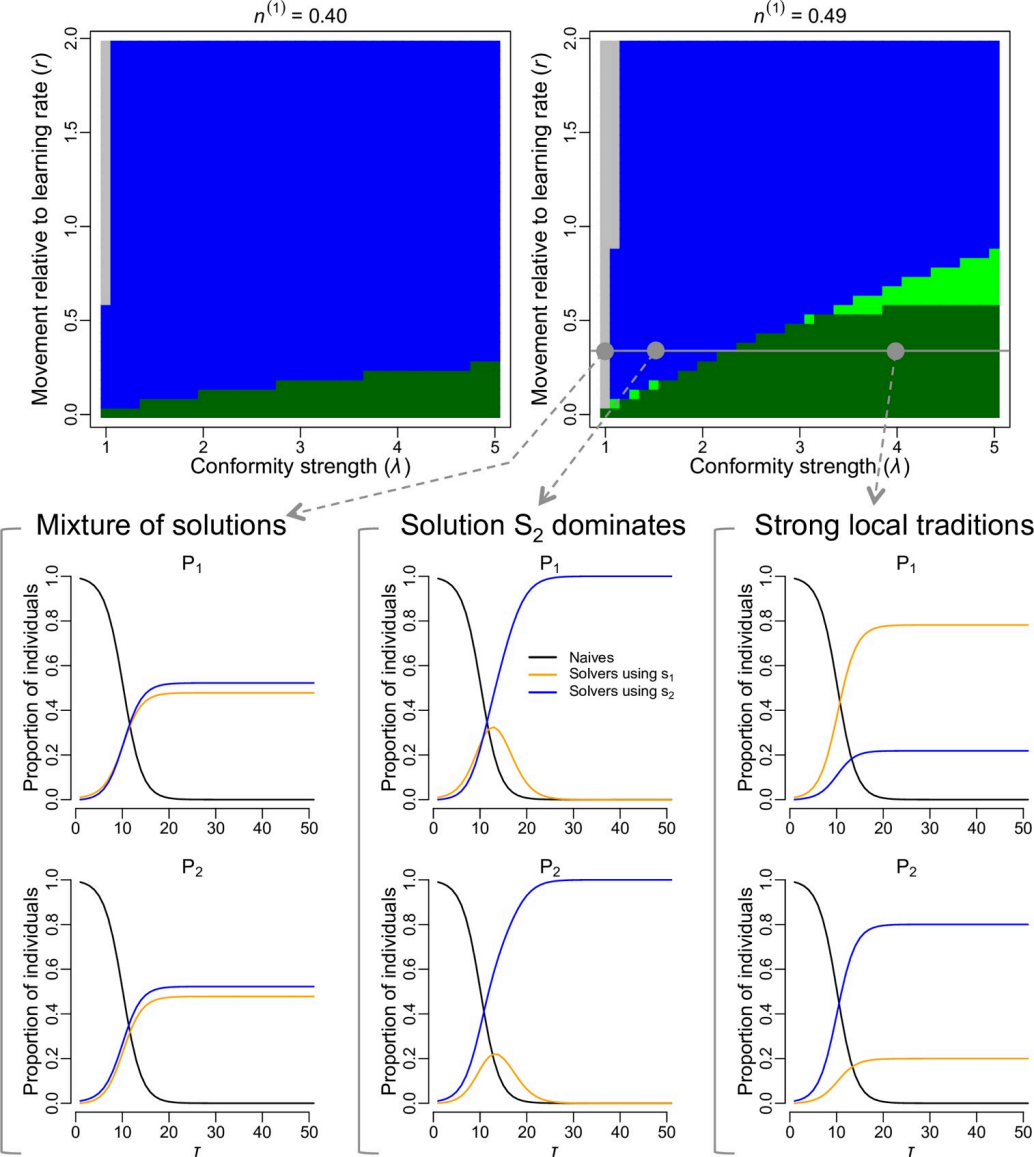
Local traditions emerge when conformity is strong relative to the movement rate. This panel shows the model outputs for the baseline model with two sub-populations. At the start of every simulation, sub-population P_1_ contained innovators using solution s_1_ (1% of its population) while sub-population P_2_ contained innovators using solution s_2_ (also 1% of its population). The phase diagrams represent different configurations of sub-population sizes, with n^(k)^ indicating the proportion of the total population size in the system existing in sub-population P_k_. Each pixel in the phase diagrams derives from a simulation run with the corresponding parameter values, and the colour of the pixel indicates the emerging pattern after 150 days: grey = mixture of solutions in every sub-population, blue = solution s_2_ dominated the whole system, light green = weak local traditions, dark green = strong local traditions (see section *Analysing emerging patterns* in Materials and Methods for details). Three examples of the evolution of the proportion of naïve individuals (black curve) and proportion of solvers using solution s_1_ (orange curve) and solution s_2_ (blue curve) in each sub-population, are shown for fixed movement rate relative to the learning rate (*r* = 0.35) and sub-population size configuration, but with varying conformity strength. When no conformity bias was included (λ = 1), sub-populations had a mixture of solutions; when conformity was relatively weak (λ = 1.5), solution s_2_ (which was seeded in the larger population) dominated both sub-populations; and when conformity as relatively strong (λ = 3.5), local traditions emerged.

In a two sub-population system, we found that the emergence of contrasting local traditions (i.e. the situation where, at the end of the simulation, one sub-population is dominated by individuals with one behavioural preference while the other sub-population is dominated by individuals with the alternative behavioural preference) strongly depends on the interplay between the strength of conformity and the magnitude of the rate of movement of individuals between the sub-populations relative to the learning rate, determined by parameters *λ* and *r* respectively (see details in Materials and Methods; hereafter the magnitude of movement rate relative to the learning rate will be refer to simply as *movement rate*). When *r* > 0, and conformity was not included in the model (i.e. *λ* = 1), the pattern that emerged (except when the difference in size between the two sub-populations was large) was a mixture of behavioural preferences in both sub-populations (Fig 1). When conformity was strong relative to the movement rate, local traditions established and remained stable (Fig 1). When conformity was weak relative to the movement rate, one of the two behavioural preferences dominated the whole system (Fig 1). In the latter case, which preference dominated depended on the relative sizes of the sub-populations. When the size of the sub-population in which seeded individuals with a given behavioural preference was initially introduced was larger than the size of the other sub-population, then that preference came to dominate at the end of the simulation.

### Simple environmental setting

**Fig 2:**
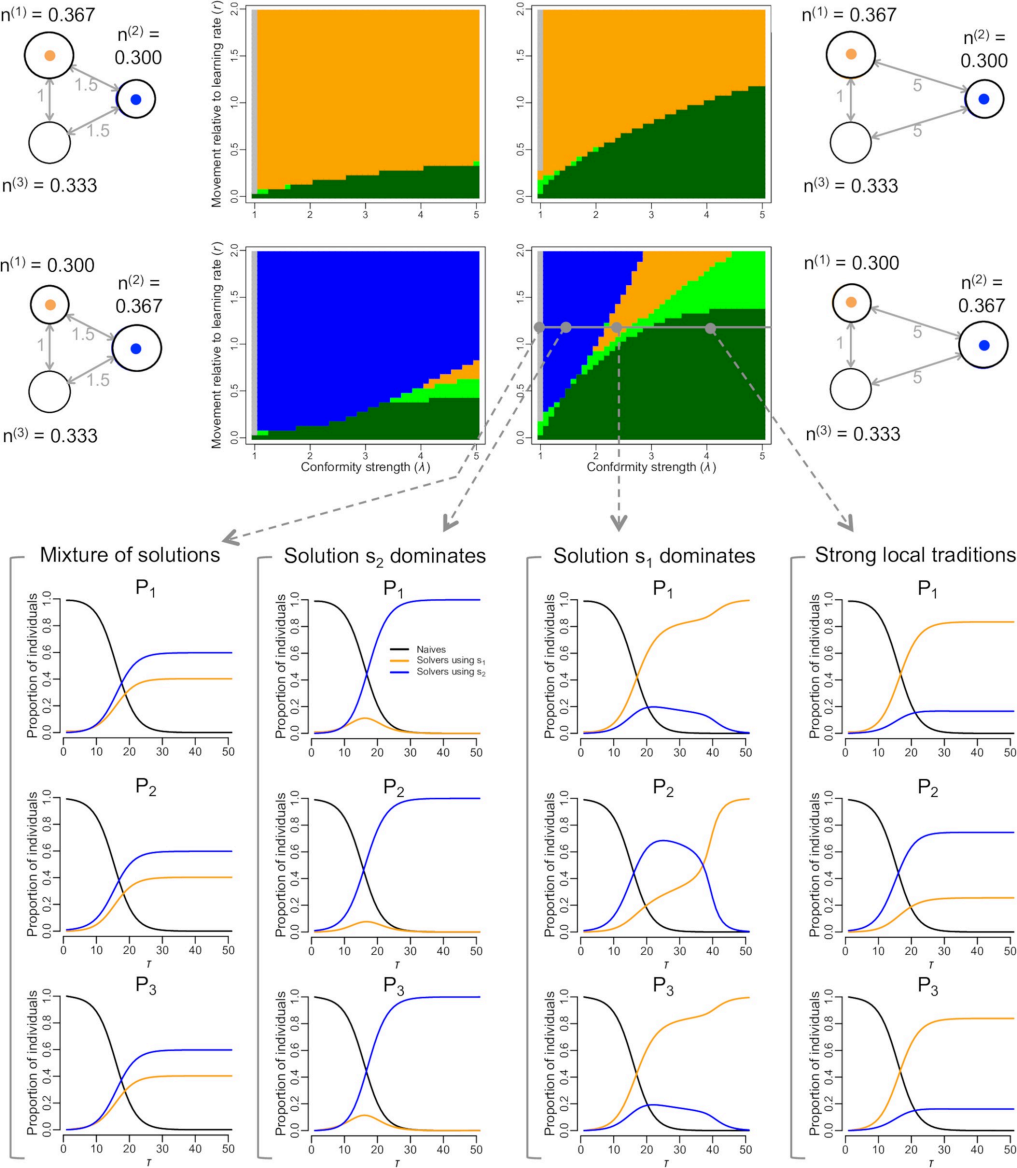
Spatial configuration of sub-populations has a complex effect on how conformity and movement lead to either the emergence of local traditions or the domination of a single solution. This figure shows the model outputs in the three-sub-population case. At the start of every simulation, sub-population P_1_ contained innovators using solution s_1_ (1% of its population), sub-population P_2_ contained innovators using solution s_2_ (also 1% of its population), and sub-population P_3_ contained only naïve individuals. Each pixel in the phase diagrams corresponds to a simulation run with the corresponding parameter values, and the colour of the pixel indicates the emerging pattern after 150 days: grey = mixture of solutions in every sub-population, blue = solution s_2_ dominated the whole system, orange = solution s_1_ dominated the whole system, light green = weak local traditions, dark green = strong local traditions. The two columns of phase diagrams represent different spatial configurations; that is different distances between sub-population 2 and the other two sub-populations (whose distance separating them was set to 1), generating different movement rates between pairs of sub-population. The two rows of phase diagrams represent a different configuration of sub-population sizes, with n^(k)^ indicating the proportion of the total population size in the system that occur in sub-population P_k_. Four examples of the evolution of the proportion of naïve individuals (black curve) and proportion of solvers using solution s_1_ (orange curve) and solution s_2_ (blue curve) in each sub-population, are shown for fixed movement rate relative to the learning rate (r = 1.2), and spatial and sub-population sizes configuration, but with varying conformity strength. When no conformity bias was included (λ = 1), sub-population contained a mixture of solutions; when conformity was relatively weak (λ = 2), solution s_2_ ended up dominating in every sub-population; when conformity was intermediate (λ = 3), solution s_1_ ended up dominating in every sub-population; and when conformity was relatively strong (λ = 4), local traditions emerged.

To examine the role of space in the spread of behavioural preferences, we extended the baseline model to a simple environmental setting with three spatially distinct sub-populations, thereby effectively adding an extra sub-population containing no innovators at the start of the simulation (see Materials and Methods for details). Once again, when conformity was not included in the model (i.e, *λ* = 1), the pattern that always emerged regardless of other parameter values was a mixture of behavioural preferences in all sub-populations (Fig 2). Similarly to the baseline model, there was a strong interplay between movement rate and conformity strength; when conformity was strong relative to the movement rate, local traditions established and were stable (i.e. two sub-populations were dominated by individuals with one behavioural preference while the third sub-population was dominated by individuals with the alternative behavioural preference; Fig 2), and when conformity was weak relative to the movement rate, one of the two behavioural preferences ultimately dominated the entire system (Fig 2). In the latter case, which preference dominated depended on both the relative sizes of the sub-populations as well as their spatial configuration (i.e. the distances separating the sub-populations, which determines the relative movement rates of individuals between pairs of sub-populations). When sub-populations were equidistant, the behavioural preference initially introduced in the largest sub-population ended up dominating the system. Increasing the distance separating a sub-population in which innovators with a given behavioural preference were initially introduced from the two other sub-populations (i.e. creating unequal movement of individuals between sub-populations) resulted in this preference not being able to dominate the system at the end of the simulation (S3 Fig).

Increasing the distance between sub-populations also affected how much stronger/weaker conformity must be in comparison to the movement rate to generate the emerging patterns: the smaller the distance (i.e. the greater the movement of individuals), the stronger conformity had to be for local traditions to establish and stabilise (Fig 2 and S3 Fig). When difference in sub-population size and spatial configuration acted in opposite directions, then either behavioural preference could ultimately dominate, and the outcome depended on the interplay between the strength of conformity and the magnitude of the movement rate. A behavioural preference generally became dominant when the sub-population in which it was introduced was larger than the sub-population into which the alternative behavioural preference was introduced (Fig 2). When the larger sub-population was also located relatively far from the two other sub-populations, and conformity was relatively strong but not so strong as to generate local traditions, then the behavioural preference that was released in the smaller sub-population eventually dominated the system (Fig 2).

Some unexpected results were observed when innovators were initially released in sub-populations of the same size, where both were larger than the third sub-population, but one of them was located further away. In this case, the behavioural preference initially introduced in the most distant sub-population ended up dominating the system (S3 Fig, second plot of fifth row, in blue). This arose when conformity was very weak compared to the movement rate, and it contrasts with what might have been expected based on other results, which predict that the nearest of the two sub-populations should dominate.

### Realistic environmental setting

**Fig 3:**
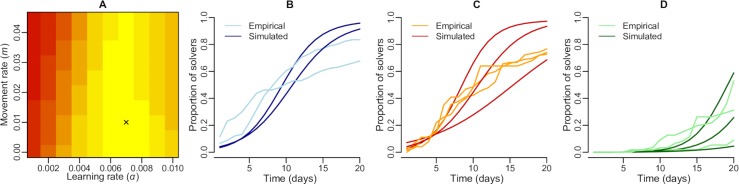
Scan of parameter space and model calibration. A: Scan of parameter space over the learning rate α and the movement rate *m*. The darker red colours indicate higher sum of squares between empirical and simulated diffusion curves. The black cross indicate the parameter values generating the lowest sum of squares between empirical and simulated diffusion curves, thus indicating the model that is best able to replicate these diffusion curves (i.e. *best-fit model*). B: Empirical and simulated (for the best-fit model) diffusion curves over 20 days for the sub-populations in which solvers using solution s_2_ where initially released; C: Empirical and simulated (for the best-fit model) diffusion curves over 20 days for the sub-populations in which solvers using solution s_1_ where initially released; D: Empirical and simulated (for the best-fit model) diffusion curves over 20 days for the control sub-populations in which no trained solvers where initially released.

**Fig 4:**
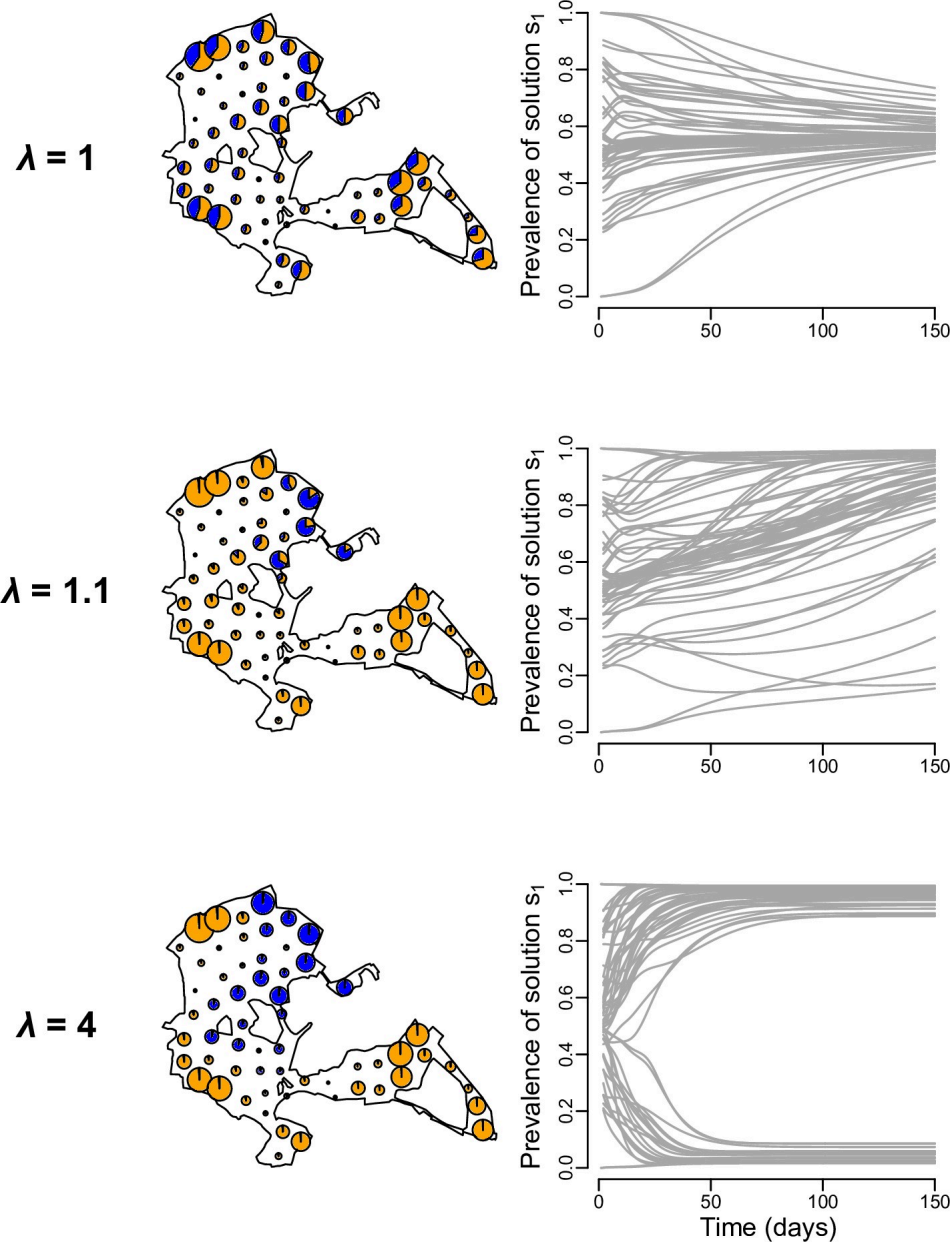
Predictions for the spread of information in a great tit cultural diffusion experiment in Wytham Woods. This analysis was designed to replicate the conditions of the cultural diffusion experiment performed by Aplin et al. [11], in which two alternative foraging techniques were introduced in the great tits population of Wytham Woods, United Kingdom, and their spread through time monitored. Three examples of the evolution of the prevalence of solution s_1_ among solvers are shown for the movement rate (m = 0.01) and learning rate (α = 0.007) corresponding to the model best able the replicate the empirical diffusion curves (Fig 3), but with varying conformity strength. The maps represent the extent of Wytham Woods and the location of the 60 internally sited feeders. Each feeder is represented by a pie chart indicating the number of naïve individuals (grey), solvers using solution s_1_ (orange) and solvers using solution s_2_ (blue), and the size of the pie chart is proportional to the total number individuals occurring around the feeder (i.e. the size of the sub-population). The right column shows the evolution of the prevalence of solution s_1_ among solvers at each feeder (each grey line represents a feeder). When no conformity bias was included (λ = 1) the model predicted a mixture of solutions in every sub-population; when conformity was relatively weak (λ = 1.1) the system tended to converge towards solution s_1_ dominating; and when conformity was relatively strong (λ = 4) local traditions emerged.

To examine the role of habitat structure and the ecological process of movement in a realistic setting, we extended the model to represent the great tit population of Wytham Woods, near Oxford, which has been the subject of a long-running study, and the site of a recent cultural diffusion experiment [11]. The model of spread of behavioural preference for this real-world population in a natural environment (see Materials and Methods for details) was calibrated by comparing simulated and empirical diffusion curves in sub-populations that were monitored by Aplin et al. [11] (Fig 3A). The model that best matched empirical diffusion curves had a value for the parameter determining the magnitude of movement rate m = 0.01 and a learning rate α = 0.007 (hereafter termed *best-fit model*) and was able to realistically generate these diffusion curves (Fig 3). The conformity parameter *λ* did not affect the diffusion curves generated by the model. This parameter was therefore subsequently explored using the best-fit model for m and α (Fig 4).

The emerging patterns in the realistic environmental setting were consistent with those for the baseline model and its extension to three sub-populations. Three possible patterns emerged at the end of the simulation depending on parameter values: (1) a mixture of behavioural preferences in every sub-population when conformity was not included in the model (i.e. *λ* = 1), (2) domination of one behavioural preference across the entire population when conformity was weak relative to the magnitude of the movement rate, and (3) the establishment of local traditions when conformity was strong relative to the magnitude of the movement rate (i.e. some sub-populations were dominated by individuals with one behavioural preference while the rest were dominated by individuals with the alternative preference, Fig 4). Increasing the learning rate affected how much stronger/weaker conformity must be compared to the magnitude of the movement rate to generate the different emerging patterns (Fig 4). That is, for local traditions to establish and stabilise, conformity had to be stronger relative to movement rates when the learning rate was slower (Fig 4).

**Fig 5:**
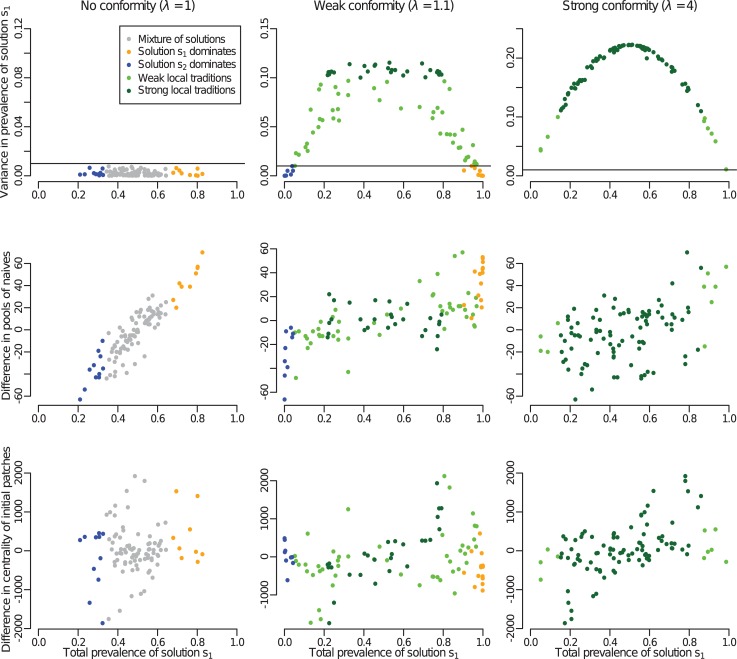
The outcome of the spread of information is sensitive to the initial conditions. This panel shows results for the randomisation of initial conditions for the spread of information in Wytham Woods. Each column of plots corresponds to a different conformity strength, for which 100 simulations with randomised initial conditions were run. In all the plots, each point corresponds to a model run with randomised initial conditions (i.e. where the trained innovators were introduced into the population at random locations). In each column, the same model runs are used in all three panels, and the colour indicates the type of pattern that emerges, which is indicated in the legend in the top left panel. (*Top row*) Values for the two summary statistics used to identify the emerging pattern. The colour of each point is based on where it is located in this plot. The horizontal line indicates the threshold above which local traditions were said to have emerged, and strong local traditions are said to have emerged above 0.1 for the variance of prevalence in solution s_1_. Below the horizontal line, if the total prevalence in solution s_1_ was below 0.33 then it is categorised as “solution s_2_ dominates” and if it was above 0.66 then it is categorised as “solution s_1_ dominates”. (*Middle row*) Relationship between the total prevalence of solution s_1_ (one of the two summary statistics) and the difference between the pool of naïve individuals initially in contact with solution s_1_ and s_2_ (i.e. size of the sub-population in which innovators with solution s_1_ were released at the start of the simulation minus the size of the sub-population in which innovators with solution s_2_ were released). (*Bottom row*) Relationship between the total prevalence of solution s_1_ and the difference in the centrality of the sub-populations in which innovators with solutions s_1_ and s_2_ were released at the start of the simulation. The centrality of a sub-population was computed as the median distance between itself and other sub-populations (the smaller the value the more central is the sub-population). The smaller the difference in centrality, the more solution s_1_ was released in a central location compared to solution s_2_.

The pattern of distribution of behavioural variants across the whole population was affected by where innovators with preferences for solutions s_1_ and s_2_ were released initially, particularly when conformity was weak relative to the magnitude of the movement rate (Fig 5). When this was the case (i.e. *λ* = 1.1), 76% of simulations in which initial conditions were randomised resulted in the emergence of local traditions, and the rest of the simulations resulted in one behavioural preference dominating the whole system (with some simulations leading to solution s_1_ to be predominant, and some simulations with solution s_2_ dominating; Fig 5). Which behavioural preference dominated was strongly affected by the sizes of the pools of naïve individuals in contact with innovators preferring each solution at the start of the simulation. If one behavioural preference came to dominate the whole system, then it was likely to be the preference that was initially added in a comparatively larger sub-population (Fig 5), consistent with previous results. However, when conformity was strong relative to the magnitude of movement rate (i.e. *λ* = 4), all of the simulations resulted in the emergence of local traditions. When no conformity was included (i.e. *λ* = 1), 80% of simulations resulted in a mixture of behavioural preferences in every sub-population, and the rest of the simulations resulted in one behavioural preference dominating the whole system (with some simulations leading to solution s_1_ throughout the landscape, and some simulations with solution s_2_ predominant).

## Discussion

Our results demonstrate the importance of the interplay between movement and conformity for determining whether or not local traditions can become established in animal populations. First, our model indicates that a conformist bias in learning is key for the emergence of local traditions, as our simulations in which a conformist bias was not included almost never led to local traditions (except when the movement rate was equals to zero or very low; Figs 1, 2, 4 and 5). The importance of conformity in this scenario is in line with previous hypotheses and indications from studies that have experimentally seeded novel information in populations [11,12,17,19]. Second, we extended this finding to show that local traditions establish only when conformity is relatively strong compared to the magnitude of the movement rate of individuals between sub-populations. This was observed for simple populations containing two (Fig 1) or three (Fig 2) sub-populations, as well as in the real environmental setting (Fig 4). When conformity was weak relative to the magnitude of the movement rate, moving individuals could continuously invade a sub-population with alternative behavioural preferences at a faster rate than that at which they could conform to the local behavioural preference in that sub-population, thereby leading to the domination of a single behavioural preference across the whole system by the end of the simulation (e.g. Figs 1, 2 and 4). Since neither of the two alternative behavioural preferences had a selective advantage, the solution that ended up dominating was determined by the initial conditions: a given behavioural preference that started in a larger pool of naïve individuals was more likely to dominate (Figs 1, 2 and 5). This is because it spread more quickly at the start of the simulation than the alternative preference, and knowledgeable individuals moving out of that sub-population therefore represented a relatively large proportion of knowledgeable individuals in each sub-population that they arrived in. Our results therefore emphasise that both the strength of the conformity bias in learning and the magnitude of the rate of movement relative to the learning rate matter for determining the pattern of spread of information and culture.

Our model is very general and can be used to make predictions for any population with knowledge of the relative size of the sub-populations, their connectivity, and the initial proportion of individuals that are innovators. The absolute total population size and the absolute size of the sub-populations, but also the absolute number of innovators of each type present in a subpopulation, do not matter here for predicting the resulting pattern of behavioural preferences across a landscape. It is their relative/proportional values that are important for determining the overall dynamics of the model.

The spatial configuration of sub-populations has an important effect on the outcome of the spread of information. Increasing habitat fragmentation, as in the three-sub-population case, led to more favourable conditions for the establishment of local traditions by lowering the movement rate and thus increasing the relative impact of conformist learning (Fig 2). The spatial configuration of sub-populations also affects which of the two alternative behavioural preferences ultimately dominates when conformity is weak relative to the magnitude of the movement rate. In the three-sub-population setting, the behavioural preference that colonises the third sub-population first (in which no innovators were introduced) is generally the one that ends up dominating at the end. This effect is determined by how far the sub-populations are from each other and the relative sizes of the sub-populations. Interestingly, if the sub-population with the largest pool of naïve individuals at the start of the simulation is also more distant from the other sub-populations, which preference predominates at the end depends on the interplay between conformity strength and the magnitude of movement rate (Fig 2 second row of phase diagrams), as this affects which preference is more likely to be the first to colonise the sub-population with no innovator.

Finally, a surprising effect of space was observed in a three-sub-population landscape in which innovators were initially introduced in two large sub-populations but where one of the two large sub-populations was located further away from the other two sub-populations. In this case, the behavioural preference of the innovator in the most distant sub-population ended up dominating the whole system when conformity was very weak relative to the magnitude of the movement rate (S3 Fig, second plot of fifth row, in blue). A possible explanation for this result is that, with a very high movement rate relative to conformity strength, naïve individuals from the sub-population without innovators move *en masse*, and therefore slow down the initial spread of the behavioural preferences. This effect was less pronounced for the preference introduced in the most distant sub-population (as movement was dependent on distance) and so this preference could subsequently colonise the sub-population without innovators faster than the alternative preference. Overall, these results highlight the important effects of habitat configuration and fragmentation on the spread of culture in animal populations (see also [20]), and allow for testable predictions to be made. It is particularly relevant given the wide range of animal populations around the world that are affected by habitat fragmentation [21,22].

Our model replicated well the diffusion curves empirically observed in a cultural diffusion experiment in great tits in Wytham Woods (Fig 3). With the same initial conditions in our model as in the field experiment (i.e. trained innovators released at the same locations in the landscape), the model was able to predict the qualitative difference between the diffusion curves over 20 days in sub-populations in which trained individuals were initially released and control sub-populations (i.e. without trained individuals initially released; Fig 3). This set of results supports the potential for this model to be used to make predictions about when novel behaviours could result in local traditions. Such predictions could, in turn, be tested in cultural diffusion experiments.

Our model predicts that local traditions are most likely to become established when the movement rate of individuals between sub-populations is low relative to the strength of conformity. If movement rates are relatively high, the location of the sub-populations in which the different behaviours first emerge has an important effect on the outcome. The model predicts that the centrality of location in the landscape largely does not affect the outcome, but that the size of the local pool of naïve individuals has an effect (Fig 5). If a weak conformist bias exists in the transmission of information, local traditions are more likely to establish and be well pronounced if two different behavioural preferences appear in sub-populations with similar sizes (Fig 5). These predictions have many implications for studying the emergence of behavioural traditions in animal populations in which social learning occurs. For example, they highlight the key and often neglected role of movement, and particularly its interplay with conformist learning, as well as the importance of the initial conditions. It should therefore be interesting going forwards to test these model predictions for species with different levels of social mobility–e.g. high mobility fission-fusion bird populations [23] versus low inter-group movement rates typified by primates [e.g. 12]–and for various initial conditions. At the same time, it will be fruitful to gain an improved understanding of the factors that shape the movement of animals through space and ultimately determine connectivity among sub-populations [14].

In this study, we modelled a scenario in which two alternative behavioural preferences were introduced at the same time into a population of naïve individuals. This is consistent with cultural diffusion experiments. However, in natural settings, it is also likely that solutions to a foraging task might be discovered and rediscovered through repeated innovations [18]. Incorporating an asocial learning rate, whereby individuals can spontaneously learn to solve the problem using a certain solution, would be an interesting, and relatively straightforward, addition to our model. However, it should have very little impact unless it is large relative to the social learning rate. Future research could also extend our model to reflect other characteristics. For example, including demographic processes could be a fruitful avenue for making long-term predictions. We assumed that the total carrying capacity of the environment had been reached and that each sub-population had a constant number of individuals. However, including varying population sizes could be interesting for exploring whether or not local traditions remain stable across multiple generations. Furthermore, the model may also be useful for considering how individual-level differences interact with the emergence and spread of culture. For example, juveniles could potentially learn faster than adults, or conformity could vary across age classes [19]. Individual-level differences have recently been highlighted as being important in shaping the dynamics of collective behaviour in animal groups [24,25]. It is therefore likely that such differences could play a major role in shaping the spread of behaviours and the establishment of local traditions in natural populations. It would also be interesting to consider a stochastic version of our model, since random events soon after traditions arrive in a naïve population may play an important role in determining the tradition that ends up dominating.

In summary, our model generates new insights into the process of cultural diffusion in animal populations, and specifically highlights the importance of the interplay between the movement of individuals and conformist learning on the emergence of animal cultures. By simply incorporating these two processes, our model is able to make predictions about the emergence and stability of local traditions, and allows the influence of quantities such as initial population conditions and the degree of habitat fragmentation to be tested. A major strength of the model is its generalisability. Future research could extend the model to explore the spread of animal culture for more than two behavioural preferences, other environmental settings and different time scales, and integrate individual differences and non-static sub-population demographics. Such exploration of the spread of socially-transmitted information in animal populations has the potential to provide additional insights into the conditions under which local traditions emerge and persist.

## Materials and methods

### Model overview

The spatially-explicit model describing the spread of animal culture integrates two processes: (1) transmission of information between individuals with a conformity bias, and (2) movement of individuals between spatially distinct sub-populations of habitat. A novel behaviour, which consists of two equally difficult, and equally rewarding, solutions to a novel foraging resource (*s*_*1*_ and *s*_*2*_) is introduced into a population of naïve individuals (who are unable to solve the task at the time of introduction) by adding innovators, who are individuals that know how to solve the task with a preference for either solution *s*_*1*_ or *s*_*2*_. The information about how to solve the novel task, along with the preference for either one of the two alternative solutions, can be socially transmitted to other individuals. The spread of the two behavioural preferences in the population is then modelled, with simulations being run for 150 days (with a daily time step). At any time, an individual is either naïve, a solver *s*_*1*,_ or a solver *s*_*2*_. During encounters with other individuals, naïve individuals can learn from solvers, and in doing so copy their behavioural preference. When a conformity bias is included, individuals use information about the behaviour of all other individuals in the sub-population, and they are disproportionally likely to copy the most common behavioural preference among local solvers.

### The baseline model

In the initial baseline version of the model, the environment is assumed to consist of two sub-populations. Within each sub-population, we assume individuals mix entirely at random. The size of each sub-population is at equilibrium throughout the simulation (i.e. no variation during the 150 days), essentially assuming that each sub-population is at its strict carrying capacity. In each sub-population *j* (where *j* = 1 or *j* = 2), the change of the numbers of individuals that are naïve (*U*^(*j*)^), solvers with a preference for solution *s*_*1*_ ($S_{1}^{\left(j\right)}$) and solvers with a preference for solution *s*_*2*_ ($S_{2}^{\left(j\right)}$) through time is modelled using a system of differential equations. The change in the composition of individuals in sub-population *j* = 1 is given by:

$$\left\{ \begin{array}{l} \;\frac{dU^{\left(1\right)}}{dt}=-\alpha (S_{1}^{\left(1\right)}+S_{2}^{\left(1\right)})U^{\left(1\right)}-\frac{m({U^{\left(2\right)}+S}_{1}^{\left(2\right)}+S_{2}^{\left(2\right)})U^{\left(1\right)}}{d}+\frac{m({U^{\left(1\right)}+S}_{1}^{\left(1\right)}+S_{2}^{\left(1\right)})U^{\left(2\right)}}{d}\\ \frac{d{S_{1}}^{\left(1\right)}}{dt}=L_{S_{1}}^{\left(1\right)}\alpha ({S_{1}}^{\left(1\right)}+{S_{2}}^{\left(1\right)})U^{\left(1\right)}-L_{S_{2}}^{\left(1\right)}\alpha ({S_{1}}^{\left(1\right)}+{S_{2}}^{\left(1\right)}){S_{1}}^{\left(1\right)}+L_{S_{1}}^{\left(1\right)}\alpha ({S_{1}}^{\left(1\right)}+{S_{2}}^{\left(1\right)}){S_{2}}^{\left(1\right)}-\frac{m(U^{\left(2\right)}+{S_{1}}^{\left(2\right)}+{S_{2}}^{\left(2\right)}){S_{1}}^{\left(1\right)}}{d}+\frac{m(U^{\left(1\right)}+{S_{1}}^{\left(1\right)}+{S_{2}}^{\left(1\right)}){S_{1}}^{\left(2\right)}}{d}\\ \frac{d{S_{2}}^{\left(1\right)}}{dt}=L_{S_{2}}^{\left(1\right)}\alpha ({S_{1}}^{\left(1\right)}+{S_{2}}^{\left(1\right)})U^{\left(1\right)}-L_{S_{1}}^{\left(1\right)}\alpha ({S_{1}}^{\left(1\right)}+{S_{2}}^{\left(1\right)}){S_{2}}^{\left(1\right)}+L_{S_{2}}^{\left(1\right)}\alpha ({S_{1}}^{\left(1\right)}+{S_{2}}^{\left(1\right)}){S_{1}}^{\left(1\right)}-\frac{m(U^{\left(2\right)}+{S_{1}}^{\left(2\right)}+{S_{2}}^{\left(2\right)}){S_{2}}^{\left(1\right)}}{d}+\frac{m(U^{\left(1\right)}+{S_{1}}^{\left(1\right)}+{S_{2}}^{\left(1\right)}){S_{2}}^{\left(2\right)}}{d} \end{array}\right.$$

Following some algebra it can be shown that these three equations sum to zero. The equations for *j* = 2 are similar. In the equations above, the rightmost terms describe the movement rate as a decreasing function of the distance *d* separating the sub-populations, with a parameter *m* determining the magnitude of the movement rate between the sub-populations. To maintain the size of the sub-populations at constant values (i.e. at carrying capacity), the movement rate of individuals was modelled as proportional to the number of individuals in the destination sub-population. The leftmost terms on the right-hand side of the equations describe the learning process, with some naïve individuals becoming solvers and some solvers changing preference for a solution to the problem. The magnitude of the learning rate is governed by the parameter *α*, and the rate at which naïve individuals acquire each one of the two alternative solutions (*s*_*1*_ and *s*_*2*_) is a function of the proportion of solvers with this behavioural preference among all the solvers in the local sub-population. $L_{S_{1}}^{\left(1\right)}$ and $L_{S_{2}}^{\left(1\right)}$ correspond to the conformist learning functions for learning solutions *s*_*1*_ and *s*_*2*_ respectively. These terms are defined using a generic function of the prevalence of solution *s*_*1*_ in the sub-population (*x*; i.e. proportion of individuals using solution *s*_*1*_ among solvers in local sub-population) and the parameter *λ*:

$$f_{\lambda }(x)={\left[{\left(x^{-1}-1\right)}^{\lambda }+1\right]}^{-1}$$

If *P*^(1)^ is the proportion of individuals using solution *s*_*1*_ among solvers in sub-population 1, i.e. $P^{\left(1\right)}=\frac{{S_{1}}^{\left(1\right)}}{{S_{1}}^{\left(1\right)}+{S_{2}}^{\left(1\right)}}$, then we have:

$$L_{S_{1}}^{\left(1\right)}=f_{\lambda }(P^{\left(1\right)})$$

$$L_{S_{2}}^{\left(1\right)}=1-L_{S_{1}}^{\left(1\right)}$$

These conformist learning functions produce a sigmoidal relationship between a solution’s prevalence in the sub-population and the probability of adoption of that behavioural preference (called acquisition curve; [26,27]; S1 Fig). The conformity parameter *λ* determines the strength of sigmoidality (i.e. S-shapedness) of the acquisition curve. If *λ* = 1, there is no conformity bias included in the model (i.e. straight 1:1 line; see S1 Fig). Conformist learning (from naïve to solver) and conformist switching (from solving the problem using one solution to using the alternative solution) were modelled in the same way using the same parameters. By doing this, the likelihood of an individual learning from another is approximately independent of whether or not the individual already has a preference for one of the solutions to the problem. When 0 < *λ* < 1, the least common solution to the problem is more likely to be copied than its prevalence and the dominant solution is less likely to be copied than its prevalence (S2 Fig), while when *λ* = 0, the two solutions are equally likely to be copied regardless of their respective prevalence (S2 Fig).

At the start of each simulation, innovators (i.e. knowledgeable individuals) with solution *s*_*1*_ were added to one sub-population and innovators with solution *s*_*2*_ were added to the other sub-population. We ran the model for various conformity strengths and movement rate magnitudes. As there are only two sub-populations here (1% of its population), changing the distance between the sub-populations (1% of its population) is equivalent to changing the movement rate (see equations above), so we therefore set *d* = 1 for every simulation run. To investigate the effect of sub-population size, we also ran the model with different numbers of naïve individuals in each sub-population at the start of the simulation (see Fig 1 and S2 Fig).

### Generalised form of the model

The system of equations presented above for the baseline model with two sub-populations can be simplified and generalised. Since sub-population sizes, *N*^(*j*)^, are assumed constant, $U^{\left(j\right)}=N^{\left(j\right)}-(S_{1}^{\left(j\right)}+S_{2}^{\left(j\right)})$. The system can therefore be fully described with ${dS}_{1}^{\left(j\right)}/dt$ and ${dS}_{2}^{\left(j\right)}/dt$. Moreover, time, which is meaningless in the theoretical cases investigated here, can be rescaled by the learning rate in the model, essentially eliminating *α*. To do so, we introduce *τ* = *αt*, with *t* denoting absolute time, as well as *r* = *m*/*α*, which denotes the rate of movement relative to the learning rate. The latter determines the qualitative behaviour of the system, rather than the individual values of the movement or learning rates. Furthermore, each equation can be rescaled by the absolute size of the corresponding sub-population. This makes the model more general as results can be expected to hold for any population with the same distribution of sub-population structure, regardless of its total size. It also emphasizes that this is the relative, rather than absolute, size of each subpopulation that matters for our analysis. To do this rescaling, let $s_{k}^{\left(j\right)}=S_{k}^{\left(j\right)}/N^{\left(j\right)}$, with *k* being one of the two solutions to the problem; $n_{s}^{\left(j\right)}=s_{1}^{\left(j\right)}+s_{2}^{\left(j\right)}$, with $n_{s}^{\left(j\right)}$ therefore describing the proportion of solvers in sub-population *j*; and *n*^(*j*)^ = *N*^(*j*)^/*N*, where *N* = ∑_*j*_*N*^(*j*)^ is the total population size. Implementing these simplifications and generalisations, the system of equations becomes

$$\frac{d{s_{k}}^{\left(j\right)}}{d\tau }=n^{\left(j\right)}{n_{s}}^{\left(j\right)}\left[f_{\lambda }(\frac{{s_{k}}^{\left(j\right)}}{{n_{s}}^{\left(j\right)}})-{s_{k}}^{\left(j\right)}\right]+r\sum_{i\neq j}\frac{n^{\left(i\right)}}{d_{ji}}({s_{k}}^{\left(i\right)}-{s_{k}}^{\left(j\right)})$$

where *d*_*ji*_ is the distance between sub-population *j* and *i*.

### Single population analysis

We explored the case of a single sub-population, essentially removing spatial structure in the interactions between individuals as well as movement. In this case the equation governing the dynamics of the system is

$$\frac{ds_{k}}{d\tau }=n_{s}\left[f_{\lambda }(\frac{s_{k}}{n_{s}})-s_{k}\right]$$

This case has two equations (i.e. for *k* = 1 and *k* = 2) and one parameter, *λ*, determining the strength of conformity. It is therefore possible to investigate the full phase portrait, as well as investigate how *f*_*λ*_ behaves and its consequence for within-subpopulation dynamics.

### Simple environmental setting

We extended the baseline model to three sub-populations. At the start of each simulation, innovators with solution *s*_*1*_ were added to one sub-population and innovators with solution *s*_*2*_ were added to another sub-population (the third sub-population (1% of its population) consisted of only naïve individuals). We ran simulations for the same ranges of values of conformity strength (*λ*) and magnitude of the movement rate relative to learning rate (*r*) as for the baseline model. We also investigated the effect of sub-population (1% of its population) size by running simulations with the different numbers of naïve individuals in each sub-population at the start of the simulation (see Fig 2 and S3 Fig). To investigate the effect of habitat fragmentation, we varied the distance between the sub-population where individuals trained to solve the problem with solution *s*_*2*_ were added and the two other sub-populations, investigating distances 1, 1.5 and 5, while the distance separating the two other sub-populations was maintained at 1 (see schematics in Fig 2).

### Realistic environmental setting

Wytham Woods, Oxfordshire, UK (51° 46’ N, 01° 20’ W) is a 385ha broadleaf deciduous woodland surrounded by open farmland and covered by an evenly-spaced grid of 60 feeders (see S4 Fig). This is the location where Aplin et al. [11] performed the cultural diffusion experiment in great tits, introducing alternative novel foraging techniques and monitoring their spread. We extended the baseline model to this realistic setting by adapting the non-generalised form of the model (see S1 Text) in order to replicate the same conditions as in the field study. We started simulations by releasing two innovators at targeted sub-populations in a similar fashion (i.e. at the same feeders; see S4 Fig). We divided the landscape so that each sub-population in our model contained one feeder. The total number of individuals across the woods and relative sub-population size (i.e. the number of individuals in each sub-population around each feeder in each time step) were derived from data described in [13]. To measure the distance between pairs of sub-populations, we used the *forest distance*, which is computed as the length of the shortest route between the two sub-populations through the forest (without crossing open ground). This is known to be an ecologically relevant measure of distance with regard to movement within this population [13].

### Scan of parameter space

For the realistic environmental setting of Wytham Woods, the diffusion curves outputted by the model can be compared the empirical diffusion curves obtained by Aplin et al. [11]. Two parameters are expected to significantly affect the evolution of the proportion of solvers in local sub-populations, the magnitude of the movement rate (*m*) and the learning rate (*α*). We conducted a coarse scan of the parameter space for these two parameters (see Fig 3A), investigating the following range of values: *m* ∈ [0,0.05] and *α* ∈ [0,0.01]. For each model run, we computed the sum of squares between the simulated and empirical diffusion curves (i.e. summing the square of the difference each day during the first 20 days for the 8 locations monitored in Aplin et al. [11], which are indicated in S4 Fig). We selected the model with the set of parameter values leading to the minimal total sum of squares, indicating a best match between simulated and empirical diffusion curves. These selected values for *m* and *α* were then used for all subsequent analyses.

### Sensitivity analysis

For the realistic environmental setting of Wytham Woods, we modified the initial conditions to investigate how these changes affected the model outcomes. We randomised the locations of feeders where trained individual (i.e. innovators) were released at the start of simulations. For three different values of conformity strength *λ* = 1 (no conformity included), *λ* = 1.2 (weak conformity) and *λ* = 4 (strong conformity), we ran 100 simulations, each with a random location (i.e. sub-population) where two innovators with solution *s*_*1*_, and another random location where two innovators with solution *s*_*2*,_ were added at the start.

### Analysing emerging patterns

In all model runs for every environmental setting (two sub-populations, three sub-populations and Wytham Woods), we reported the total prevalence of solution *s*_*1*_ across the whole population at the end of the simulation (*P*_*tot*_; i.e. proportion of individuals with behavioural preference for solution *s*_*1*_ among all solvers in all sub-populations) and the spatial variance of the final prevalence of solution *s*_*1*_ in sub-populations (*P*_*var*_; i.e. variance in the proportion of individuals with behavioural preference for solution *s*_*1*_ among local solvers in each sub-population). These two summary statistics were used to identify the emerging patterns:

- if *P*_*var*_ > 0.1: *strong local traditions* established at the end of the simulation (i.e. some sub-populations are strongly dominated by one behavioural preference while the others are strongly dominated by the alternative preference)
- if 0.1 > *P*_*var*_ > 0.01: *weak local traditions* established at the end of the simulation (i.e. some sub-populations have a bit more of one behavioural preference while the others have a bit more of the alternative preference)
- if *P*_*var*_ < 0.01 and *P*_*tot*_ > 0.66: *solution s*_*1*_ *dominated* across the whole system at the end of the simulation
- if *P*_*var*_ < 0.01 and *P*_*tot*_ < 0.33: *solution s*_*2*_ *dominated* across the whole system at the end of the simulation
- if *P*_*var*_ < 0.01 and 0.33 > *P*_*tot*_ < 0.66: *mixture of solutions* in every sub-population

These criteria and thresholds were chosen in order to best reflect a visual identification of the emerging patterns (see examples in Fig 4).

## Supporting information

S1 Text Extended model to more than two patches. (PDF)

S1 Fig Sigmoidal acquisition curves indicating a conformity bias for different values of parameter *λ*. (EPS)

S2 Fig Effect of conformity on the dynamics of acquisition of behavioural preferences in a single mixed population. (*Top row*) Acquisition curves for different values of *λ*. (*Bottom row*) Phase portrait of *ds*_1_/*dτ* and *ds*_2_/*dτ* for corresponding values of *λ* (by column).
(EPS)

S3 Fig Model outputs for the environmental setting with three sub-populations. At the start of every simulation, sub-population P_1_ contained innovators using solution s_1_ (1% of its population), while sub-population P_2_ contained innovators using solution s_2_ (also 1% of its population), and sub-population P_3_ contained only naïve individuals. Each pixel in the phase diagrams corresponds to a simulation run with the corresponding parameter values, and the colour of the pixel indicates the emerging pattern after 150 days: grey = mixture of solutions in every sub-population, blue = solution s_2_ dominated the whole system, orange = solution s_1_ dominated the whole system, light green = weak local traditions, dark green = strong local traditions. The two columns of phase diagrams represent different spatial configurations: a different distance between sub-population 2 and the other two sub-populations (whose distance separating them was set to 1), which results in different relative migration rates between pairs of sub-populations. The rows of phase diagrams represent a different configuration of sub-population sizes, with n^(k)^ indicating the proportion of the total population size in the system that occur in sub-population P_k_.
(EPS)

S4 Fig Map of Wytham Woods. Each grey circle indicates the location of a feeding station, and the diameter of the circle corresponds to the number of individuals (i.e. great tits Parus major) occurring around the feeding station (i.e. a sub-population). Red stars indicate the sub-populations where two innovators trained to perform solution s1 were released at the start of simulations, and blue stars indicate the sub-populations where two innovators trained to perform solution s1 were released at the start of simulations. These locations are identical to those used in the cultural diffusion experiment performed by Aplin et al. (2015). Green stars indicate sub-populations where no trained individuals were released but in which diffusion curves were monitored in Aplin et al. (2015).
(EPS)
