# Supplementary material for: Movement and conformity interact to establish local behavioural traditions in animal populations
Source: PLoS Comput Biol. 2018 Dec 20;14(12):e1006647. doi: 10.1371/journal.pcbi.1006647 (PMC6319775; doi:10.1371/journal.pcbi.1006647)
Supplement: S1 Text — (PDF) [file pcbi.1006647.s001.pdf]

SUPPORTING INFORMATION

Supporting Methods. Extended model to more than two patches.

We extended the baseline model to *N* patches. This required modification of the equations, which were then used for the realistic environmental setting of Wytham Woods (60 patches). In the sub-population occurring in a given patch *j*, the evolution of the numbers of individuals that are solvers with a behavioural preference for solution s_1 (S_1) and solvers with a behavioural preference for solution s_2 (S_2) through time was modelled using differential equations. For example, the evolution of the numbers of individuals of each type in patch *j* = 1 was given by:

$$\begin{cases} \frac{d{S_1}^{(1)}}{dt} = L_{S_1}^{(1)}\alpha\left({S_1}^{(1)} + {S_2}^{(1)}\right)U^{(1)} - L_{S_2}^{(1)}\alpha\left({S_1}^{(1)} + {S_2}^{(1)}\right){S_1}^{(1)} + L_{S_1}^{(1)}\alpha\left({S_1}^{(1)} + {S_2}^{(1)}\right){S_2}^{(1)} - \sum_{i=2}^{N} \frac{m(S_1^{(i)} + S_2^{(i)} + U^{(i)}){S_1}^{(1)}}{d_i} + \sum_{i=2}^{N} \frac{m(S_1^{(1)} + S_2^{(1)} + U^{(1)}){S_1}^{(i)}}{d_i} \\ \frac{d{S_2}^{(1)}}{dt} = L_{S_2}^{(1)}\alpha\left({S_1}^{(1)} + {S_2}^{(1)}\right)U^{(1)} - L_{S_1}^{(1)}\alpha\left({S_1}^{(} + {S_2}^{(1)}\right){S_2}^{(1)} + L_{S_2}^{(1)}\alpha\left({S_1}^{(1)} + {S_2}^{(1)}\right){S_1}^{(1)} - \sum_{i=2}^{N} \frac{m(S_1^{(i)} + S_2^{(i)} + U^{(i)}){S_2}^{(1)}}{d_i} + \sum_{i=2}^{N} \frac{m(S_1^{(1)} + S_2^{(1)} + U^{(1)}){S_2}^{(i)}}{d_i} \end{cases}$$

with similar equations for other patches. The evolution of the number of individuals that are naïve (U) can be derived from the equations above, as subpopulation sizes, $N^{(j)}$, are known and assumed constant, and $U^{(j)} = N^{(j)} - (S_1^{(j)} + S_2^{(j)})$.

In the equations above, d_i corresponds to the distance separating patch 1 and patch *i*, and $L_{S_1}^{(1)}$ and $L_{S_2}^{(1)}$ correspond to the conformist learning functions for learning solutions s_1 and s_2 respectively in patch 1 (see details in the main text).
